# Medical students as human subjects in educational research

**DOI:** 10.3402/meo.v18i0.19524

**Published:** 2013-02-25

**Authors:** Umut Sarpel, Mary Ann Hopkins, Frederick More, Steven Yavner, Martin Pusic, Michael W. Nick, Hyuksoon Song, Rachel Ellaway, Adina L. Kalet

**Affiliations:** 1 Department of Surgery, Mount Sinai School of Medicine, New York, NY, USA; 2 Department of Surgery, New York University School of Medicine, New York, NY, USA; 3 Department of Epidemiology and Health Promotion, New York University College of Dentistry, New York, NY, USA; 4 Consortium for Research and Evaluation of Advanced Technology in Education, Steinhardt School of Culture, Education, and Human Development, New York University, New York, NY, USA; 5 Department of Emergency Medicine, New York University School of Medicine, New York, NY, USA; 6 WISE-MD Project, New York University, New York, NY, USA; 7 Department of Teacher Education, School of Education, Georgian Court University, Lakewood Township, NJ, USA; 8 Human Sciences, Northern Ontario School of Medicine, Sudbury, ON, Canada; 9 Division of Educational Informatics, Department of Medicine, New York University School of Medicine, New York, NY, USA

**Keywords:** institutional review board, educational research, computer-assisted instruction, learning, medical students

## Abstract

**Introduction:**

Special concerns often arise when medical students are themselves the subjects of education research. A recently completed large, multi-center randomized controlled trial of computer-assisted learning modules for surgical clerks provided the opportunity to explore the perceived level of risk of studies where medical students serve as human subjects by reporting on: 1) the response of Institutional Review Boards (IRBs) at seven institutions to the same study protocol; and 2) the thoughts and feelings of students across study sites about being research subjects.

**Methods:**

From July 2009 to August 2010, all third-year medical students at seven collaborating institutions were eligible to participate. Patterns of IRB review of the same protocol were compared. Participation burden was calculated in terms of the time spent interacting with the modules. Focus groups were conducted with medical students at each site. Transcripts were coded by three independent reviewers and analyzed using Atlas.ti.

**Results:**

The IRBs at the seven participating institutions granted full (n=1), expedited (n=4), or exempt (n=2) review of the WISE Trial protocol. 995 (73% of those eligible) consented to participate, and 207 (20%) of these students completed all outcome measures. The average time to complete the computer modules and associated measures was 175 min. Common themes in focus groups with participant students included the desire to contribute to medical education research, the absence of coercion to consent, and the low-risk nature of the research.

**Discussion:**

Our findings demonstrate that risk assessment and the extent of review utilized for medical education research vary among IRBs. Despite variability in the perception of risk implied by differing IRB requirements, students themselves felt education research was low risk and did not consider themselves to be vulnerable. The vast majority of eligible medical students were willing to participate as research subjects. Participants acknowledged the time demands of their participation and were readily able to withdraw when those burdens became unsustainable.

## Introduction

Institutional Review Boards (IRBs) were established in the United States in the late 1970s as one mechanism to protect human subjects in research (1). IRBs review proposed research involving human subjects to ensure that the risks to participants are minimized as much as possible, that all risks are clearly defined and transparent, that participants’ consent to participate is both informed and voluntary, and that the study serves some meaningful purpose that justifies involving human subjects.

The Code of Federal Regulations (CFR) allows an ‘exempt’ status for studies that pose low risk to its participants (2). Medical education research is an example of a field where the study methods employed (typically observations or comparisons of teaching methods) usually entail no more than minimal risks to their participants. There are particular issues arising from when students themselves are the subjects of education research (3, 4). There are three major concerns; first that medical students may feel unduly pressured to participate in such research, second that student-teacher relationships may be compromised or may compromise the research, and third that research on medical students may pose risks that are not readily apparent to the investigators or the participants.

One of the core tenets of informed consent is that potential research subjects should not in any way feel coerced to participate and that those who agree to participate are free to withdraw at any time without penalty. Consent is not voluntary when potential subjects feel pressure to participate, whether the pressure is explicit or implied. Some authors have suggested that medical students are a vulnerable population or captive audience, observing that even though recruitment to a study is presented as voluntary, students within the close-knit community of a medical school may nevertheless feel that their lack of participation might precipitate a negative response from the faculty, or in some other way prejudice their standing or progress (5, 6). Christakis points out, ‘the same ready availability that makes medical students a desirable subject pool also makes them a captive population, a status that can only decrease their autonomy’ (7).

Another area of concern regarding medical students as human subjects involves the possibility that education research may pose risks that are not readily apparent to the participants or the researchers. For instance, time-consuming research assessments may distract students from their medical studies (4). Because the risk is not in the study task itself, but in students’ lost opportunity to focus on their education, it is possible that the true risk of participation is not transparent, and therefore poses greater than minimal risk. Although there are common guidelines, lack of consensus on these topics at different institutions has led to disparate IRB evaluations of educational studies where medical students are the human subjects.

While there has been concern expressed over the risk to medical students as human subjects, there has been little published exploring medical students’ behavior when enrolled in educational research studies. The Web Initiative in Surgical Education Trial (WISE Trial) presented an opportunity to further explore these issues and challenges, faced by both researchers and learners as research subjects. The WISE Trial was a multi-institutional randomized controlled trial to evaluate the efficacy of web-based instruction as a supplemental learning aid during surgical clerkships (8). The objective of the trial was to compare the educational efficacy of instructional design enhancements among different versions of two online learning modules one on the topic of appendicitis the other on carotid stenosis. In parallel with the trial, we studied the process of trial implementation across the multiple sites, and used qualitative methods to assess student experiences as study subjects.

In this article, we explore the perceived level of risk of studies where medical students serve as human subjects by reporting on: 1) the response of IRBs at seven institutions to the same study protocol; and 2) the thoughts and feelings of students across study sites about being research subjects.

## Methods

The New York University School of Medicine (NYU) was the home site for the WISE Trial. Six other medical schools in the United States were recruited as additional study sites. The participating institutions represented diverse geography (region of the country), setting (urban and rural), institution type (private and public), and class size. The WISE Trial was conducted over one academic year, July 2009 through August 2010. All third-year medical students rotating through the required surgery clerkship at all seven collaborating institutions were eligible to participate.

The study protocol was initially submitted to the NYU IRB and underwent full board review and approval before being distributed to the other participating institutions for their local IRB review.

All students received a joint email from the WISE Trial coordinator and their surgical clerkship coordinator on the first day of their surgery clerkships, instructing them on how to log on to the study site where they could read a description of the risks, benefits, and alternatives to participation. This was followed by a brief oral introduction to the trial, typically given by the clerkship coordinator or the clerkship director. For institutions that permitted their learners to give online consent, the WISE modules were customized such that students could not access the content unless they responded to a request to participate in the study. At institutions where online consent did not meet their requirements, additional written consent was obtained. Students who declined to participate in the trial (from any institution) were provided with the standard WISE module, without the design modifications under evaluation. Local staff and faculty were not informed whether their students had consented or not. No incentives were offered for participation.

Students participating in the WISE Trial were given access to one web-based module on appendicitis and one on carotid stenosis. There were several outcome measures built into these modules: 1) a pre-test on appendicitis completed prior to using the WISE module consisting of eight questions, 2) a similar pre-test on carotid disease, 3) a post-test on appendicitis completed immediately after the module consisting of 12 multiple choice questions, 4) a similar post-test on carotid disease, 5) a mid-clerkship review covering both appendicitis and carotid disease which included of 12 multiple choice questions, and two key feature cases with 11 questions total, and 6) a final assessment at the end of rotation consisting of four key feature cases with 15 questions total on both content areas. Subjects were able to withdraw from the study at any point.

Once the WISE Trial was completed, the patterns of IRB review were compared, the participation patterns of each cohort were analyzed, and the participation burdens were calculated in terms of the time spent interacting with the modules as represented in the log files recorded by the online WISE Trial system.

After the completion of the WISE Trial, site visits were conducted at each participating institution to provide qualitative data to contextualize the quantitative study findings. Focus groups were conducted with volunteer medical students recruited by the clerkship coordinator at each site. Lunch and a $25 gift card were provided for participation in these hour-long sessions. Focus group questions covered participants’ general experiences with the WISE Trial, impressions of web-based learning, perceived risks and benefits of participation in the trial, and reasons for withdrawal from the trial. Scripted, open-ended questions were used to promote discussion and encourage all participants to contribute. One member of the WISE Trial team served as the facilitator, and another member of the team assisted by documenting the interviews with detailed notes. In order to maintain confidentiality, focus group leaders were blinded to whether or not the student volunteers had completed all sections of the trial. Sessions were audio taped and transcribed and all data were anonymized.

Three members of the team independently analyzed the transcripts using Atlas.ti, a qualitative data analysis software package (9). Inductive analysis techniques were used to code the data, develop a coding scheme, and conduct content analysis. The process of inductive analysis requires that patterns, themes, and categories of analysis come from the data, ultimately generating a coding scheme (10). Codes and sub-codes were developed to describe the topical categories to examine the key elements. Once the coding scheme was developed, the transcripts of the focus groups were reread and recoded by the same three team members. The coded transcripts were then reviewed as a group, building a consensus on the major themes identified in the analysis.

## Results

All IRBs are required to have a review and approval process, based on perceived risks to human subjects, consistent with the provisions of the Common Rule. Despite the fact that the same research protocol was submitted to all boards, one institution's IRB required a full clinical board review for approval, four institutions’ IRBs granted an expedited review, and two sites granted IRB exemption to the WISE Trial (Table 1). In addition, although online consenting was built into the WISE modules, one IRB mandated both written and online consent for their learners. This reflects considerable inter-institutional variability in the perception of risk to medical students participating in educational research.

**Table 1:** IRB requirements at medical schools participating in the WISE Trial

	Consenting students	Level of IRB review required			Consent requirement	
			
Institution	Total *N=*995	Full	Expedited	Exempt	Online	Written
A (NYU)	162	X			X	
B	22		X		X	
C	135		X		X	
D	148		X		X	
E	68		X			X
F	264			X	X	
G	196			X	X	

A total of 1,363 students were eligible for the WISE Trial, of which 995 (73%) consented to participate. Of this group, only 207 students (20%) remained enrolled for the duration of the trial over the 8–12 week clerkship and completed all outcome measures. The remaining 80% of students failed to complete one or more of the assessments.

The uninterrupted running times of the modules were 33 min for appendicitis and 47 min for carotid stenosis. For the 207 students who completed the entire trial, the median time to view the modules was 46.5 min for appendicitis, and 67.2 min for carotid stenosis. The additional time represented replaying some material or pausing the module. The average time required for completion of all assessment measures was 61.4 min; times for the individual components were as follows: appendicitis pre-test 4.4 min, carotid pre-test 5.7 min, appendicitis post-test 7.9 min, carotid post-test 8.8 min, mid-clerkship review 21.8 min, and final assessment 12.8 min. Therefore, the average time for a participant to complete both modules and all associated measures was 175 min – a little less than 3 h – over the course of their 8–12 week surgery clerkship.

Focus groups were conducted at six of the seven participating institutions. Group size ranged from four to eight students, and a total number of 35 students participated in these sessions. In focus groups, the most frequently occurring themes were 1) the desire to contribute to medical education research, 2) the absence of coercion to consent, and 3) the perception that the research constituted minimal risk. Each theme is discussed below with representative quotes from the student focus groups:

### Desire to contribute to medical education research

Focus group participants generally reported interest in participating in the WISE Trial and other medical education studies. Some expressed a sense of responsibility to participate in research, *we are medical students; we put patients through trials every day*. A number of participants also stated their willingness to be involved in the study even if it required additional time commitments: *It doesn't bother me, I understand it's part of the learning process to take a little extra time. [It's] like when we are the fourth person to come in and examine the patient for signs of an acute abdomen, it doesn't help them at all that I am poking on their belly, and making them hurt worse … but I learn a lot from being able to do that. So I'm okay with it*. Several focus group participants also stated that participation in medical education research was important to them even if they themselves did not benefit from the trial directly. *[I know there is] no benefit for me, but benefit for future iterations of this program … for next year or 2 years down the line … for other students*.

### Absence of coercion to consent

When asked if they felt obliged to participate in the trial, focus group participants widely reported that they did not feel compelled to consent. *No … I don't feel any pressure. What do you mean pressure? [Do you mean] if you say ‘no’ it looks bad or something? No, I didn't feel that*. Focus group participants were aware of the potential for coercion in medical education research, but reported that they did not feel pressured to participate. *I didn't think much about it. It seemed like a standard consent form for a research purpose, and I didn't see any harm, so I didn't think twice about it. I guess there's always a conflict … maybe some people think … [they are] being evaluated because it's part of our course … so maybe they might see a conflict there I imagine … but I don't see anything*. Respondents also reported that an important aspect of the voluntary consent process was that the clerkship director of the surgery rotation was not involved in the consenting process. Invitations to participate in the study and all subsequent correspondence were generated from the researcher's office. *So, if it was [from the clerkship director], I'm not saying I would, but I might be a little more inclined to do it. But I knew it was research, and I am familiar with you, and that you do a lot of research, so I knew more … and I did not feel like I was being graded or judged on whether I participated or not*.

### Low level of risk

In general, respondents felt the study posed little risk to them. No students voiced concerns over potential violation of privacy or loss of confidentiality. There was agreement among respondents that the only serious burden posed by participation in the study was the time demand. This reason was consistently cited as the primary reason for not persisting with the study. *At the beginning of rotations, it feels new, and you're enthusiastic about things. Towards the end, especially in surgery, you know that the shelf [exam] it's such a big percentage of your score; you're very worried about it. WISE-MD has a very long intro into each subject, and although it's very comprehensive, and it's a good review of the first-year and second-year material, a lot of times I felt like it was a lot to sit through that. So, when you're feeling pressed for time … it felt like that was kind of stressful to have to do that*. Another student stated *It's really just time … I'm so tired, I just don't want to do it*. Students reported that they were generally able to manage the time demands of the study. However, if they felt the study became too burdensome, they felt they could withdraw without repercussions. When students were asked to comment on concerns that they represent a vulnerable population, most were perplexed or incredulous: *We are vulnerable? For being a part of a web site study? What's going to hurt me? (Laughter) That's really insulting*.

## Discussion

Research that may be granted ‘exempt’ status by the IRB is defined by the Code of Federal Regulations as described below:

Exemption 45 CFR 46.101(b)(1): Research conducted in established or commonly accepted educational settings, involving normal educational practices, such as … research on the effectiveness of or the comparison among instructional techniques, curricula, or classroom management methods (2).

Exemption 45 CFR 46.101(b)(2): Research involving the use of educational tests (cognitive, diagnostic, aptitude, achievement), survey procedures, [or] interview procedures, unless … any disclosure of the human subjects’ responses outside the research could … be damaging to the subjects’ financial standing, employability, or reputation (2).

These clauses may be interpreted to allow for exemption of medical education research. However, our study found that the level of IRB review differed significantly between institutions. Despite evaluating identical research protocols, the IRBs at the seven participating institutions granted full (*n*=1), expedited (*n*=4), or exempt (*n*=2) review of the WISE Trial protocol.

Other researchers report similar variability in IRB response to identical protocols (11). Dyrbye and colleagues describe their experience submitting the identical research proposal of a medical education trial involving medical students as subjects to the IRBs at six separate medical schools. Four IRBs determined that the protocol was appropriate for expedited review, but two boards required full review (11).

This variability suggests differing assessments of the risk level of medical education research where students are the subjects. Concerns may include the idea that students within the close-knit community of a medical school might feel compelled to participate (5, 6).

Students might be subject to inappropriate and undue pressure and might participate in studies in an attempt to garner better recommendations, better grades, or other favors (such as summer employment). The rules for medical students are more stringent … because a medical student is less free than a random adult to refuse the request of a faculty investigator to be a research subject (7).

However, the qualitative data obtained from the WISE Trial focus groups did not reflect any sense that the students in the WISE Trial felt compelled to participate in the study. In fact, respondents repeatedly affirmed that they had an active desire to participate in such studies, even if they themselves did not directly benefit. Furthermore, conversations in the focus groups demonstrated that respondents did not perceive themselves as particularly vulnerable. Indeed, their readiness to withdraw from the study suggests that undue coercion to participate was not at play in this case. Importantly, our focus groups included both students who completed the trial as well as those who withdrew – therefore the opinions expressed likely illustrate both experiences.

Other published research with medical students as study subjects supports our findings. Forester et al. surveyed 524 medical students regarding their opinion on participation in medical education research (12), 93% of whom felt that medical education research was an essential part of improving their own education, and 91% reported that they did not feel coerced to participate as a subject in medical education research studies because of a faculty member's position of authority. In addition, 76% of respondents stated that they did not believe they would receive better grades, recommendations, or other favors for participating in medical education studies. Forester's group concludes that students value medical education research, want to participate as research subjects, and do not feel coerced to participate in medical education studies (12).

Another area of concern regarding medical students as research subjects is that the time demands imposed by these studies can have unforeseen risks caused by detracting students from their medical studies.

If the research would cause a medical student to lose a great deal of time from his or her courses, that research might be inappropriate. While one might argue that the student ought to determine whether it is appropriate, our perception was that the IRB also had a role in that evaluation (13).

Indeed, we did find that participation in the WISE Trial demanded significant additional time commitments and that participants were very aware of the time pressures imposed by the study. Focus group respondents frequently commented that the time demands of the study competed with the time they allotted to study for their surgical exams.

However, WISE Trial respondents also stated that these demands were usually manageable, and if the demands became too burdensome, they felt free to withdraw from the study. These findings from our focus groups support the conclusions of others who feel that medical education studies involve minimal risk to its participants so long as there is the ability to opt out.

How real a threat does research pose to a medical student's health? Probably not so great a threat as exposure to formaldehyde in gross anatomy lab or to infections incubating in a pediatric ward (14).

This study has some limitations. First, the relatively small size of the focus groups limits our ability to generalize our findings to the entire cohort. However, by design, qualitative research requires labor-intensive transcription, review, coding, and reassessment of interview transcripts. This process necessarily limits the number of participants that can be included.

Another limitation to consider is the relatively large (80%) attrition rate from the trial. The patterns and implications of attrition specific to the WISE Trial have been addressed in a separate publication (15). It is important to acknowledge that this attrition rate most likely reflects the perceived time demands imposed by participation in the trial. Studies with high attrition rates need to conduct follow-up comparisons of ‘completers’ and ‘non-completers’ and to qualify their results due to volunteer bias. For example, Callahan and colleagues demonstrated a ‘volunteer bias’ in a three decade long cohort study of medical students, demonstrating that those who do participate are more likely to be successful academically before, during and after study participation, and that underrepresented minorities and women are less likely to participate (16). This suggests that although study participation does not threaten student learning and academic success, the asymmetry in which kinds of students choose to opt out of such research threatens the validity and generalizability of its findings. This phenomena needs to be better understood and addressed in medical education research.

Another limitation is the specificity of the study intervention and the educational context. This work should also be considered in an international context. The United States is not the only jurisdiction to have IRBs, most other countries have their own similar processes but with important differences. For instance, Canada's ‘Tri-Council Policy Statement: Ethical Conduct for Research Involving Humans’ has no equivalent exemptions for educational research to those from the CFR. Further work may also need to be done to assess whether learners’ perspectives on risk with respect to research that involves them vary between countries and to what extent that may relate to the IRB processes in those jurisdictions.

In conclusion, this large multi-center trial allowed for comparison of the level of review used among IRBs when medical students are research subjects. Our findings demonstrate that risk assessment and the extent of review utilized for medical education research vary among IRBs. Despite variability in the perception of risk implied by differing IRB requirements, students themselves felt education research was low risk and did not consider themselves to be vulnerable. Participants in the WISE Trial showed that they valued medical education research and the vast majority was willing to participate as research subjects. They did not indicate any sense of coercion, and they did not seem to perceive themselves as a vulnerable population. Trial participants acknowledged the time demands of their participation in research and were readily able to withdraw when those burdens became unsustainable.

It is essential to provide thoughtful external review of medical education research in order to identify studies that have ethics issues and to provide solutions that ensure subject safety. However, greater consistency in the review process across institutions would help avoid creating disincentives to the conduct of essential educational research (11). This work is presented to stimulate further consideration of the evaluation of educational research projects by IRBs.
